# Special Issue “Advances in Spermatogenesis and Male Infertility”

**DOI:** 10.3390/ijms27198761

**Published:** 2026-09-30

**Authors:** Wan-Xi Yang

**Affiliations:** The Sperm Laboratory, College of Life Sciences, Zhejiang University, Hangzhou 310058, China; wxyang@zju.edu.cn or wxyang@spermlab.org

## 1. Introduction

Spermatogenesis is an elaborate process that contains several stages, such as the proliferation of spermatogonia, spermatocyte meiosis, spermatid differentiation, and sperm maturation. At the hormonal level, this process is controlled by the hypothalamic–pituitary–gonadal axis [1,2]; at the structural level, spermatogenesis is protected by a biological barrier in the seminiferous tubule [3]; at the gene and protein level, spermatogenesis has complex molecular regulation mechanisms, involving comprehensive signal pathways [4,5,6], Any error that occurs during this process may lead to sperm infertility, which prevents the reproduction of a Metazoan species. Research techniques are developing quickly, and our knowledge of spermatogenesis and sperm infertility is increasing.

Two review articles and eight research articles are published in this Special Issue “Advances in Spermatogenesis and Male Infertility” (Contributions 1–10), encompassing a broad range of topics.

## 2. What Aspects Can Be Highlighted in This Special Issue?

### 2.1. The Two Review Articles Broaden Our Knowledge of Sperm Flagella Diversity and Epigenetics in Spermatogenesis

In the first review, Guseva et al. introduced a systematic understanding of sperm flagella diversity in Metazoa, which contains at least 30 phyla. Although the basic morphology and structure of sperm in different phylum are similar, the ultrastructure such as the acrosome, nucleus, and flagella may differ from phylum to phylum and also from species to species. The formation of sperm flagella is very important for spermatozoa motility, and their wrong assembly causes problems. This review analyzes the diversity of the ultrastructure of sperm flagellum in Metazoa and explores the evolutionary features of the mammalian fibrous sheath proteins. This review provides a broad and in-depth understanding about the formation of different structures of sperm flagella in Metazoa. The diversity of the structure and composition of the spermatozoa fibrous sheath and the proteins involved expands our understanding of the fertility and infertility of sperm.

The second review article entitled “Unmasking the Epigenome: Insights into Testicular Cell Dynamics and Reproductive Function” was contributed by Anjum et al. Epigenetic modifications play an important role during spermatogenesis. Any abnormity in DNA methylation, histone modifications, and chromatin remodeling may lead to sperm infertility. This article comprehensively presents the role of epigenetics in spermatogenesis and reproductive health, and it emphasizes that recent advances in the epigenetic modulation of testicular function may have potential clinical application in the development of targeted diagnostic and therapeutic strategies.

### 2.2. New Discoveries of Molecules Involved in Spermatogenesis and Fertility

Three research articles introduced new knowledge about testes development and sperm quality.

Qi et al. researched how the ERK/CREB and p38 MAPK/MMP14 signaling pathways influence the spermatogenesis of *Eriocheir sinensis*, focusing on how the hemolymph–testis barrier (HTB) works in Crustacean testes. HTB is similar to the blood–testis barrier in mammalian testes, and its molecular mechanisms are not completely understood. Qi et al. found that the MAPK signaling pathway helps to maintain HTB integrity and spermatogenesis through es-MMP14 and es-CREB, which adds to our understanding of how biological barriers in different invertebrate testes work.

Zhang et al. focused on how PTSMAD4 participates in the spermatogenesis of the swimming crab *Portunus trituberculatus*. PTSMAD4 is expressed in the testes of this species, and the Smad protein is a key protein in the TGF-β signaling pathway. They determined that PTSMAD4 may be involved in the process of spermatogenesis in swimming crabs through signal transduction. Further investigation into molecular functions during spermatogenesis may benefit from this finding.

The above two articles focus on the development of crustacean testes, which is an under-researched subject. Differently, Anwar et al. investigated the relationship between the mtDNA copy number and the transcription activity of all mitochondrial protein-coding genes in young and old bulls’ spermatozoa throughout the year. They determined that there was a significant negative influence of summer and winter seasons on young bulls’ sperm-borne mitochondrial regulation.

### 2.3. Environmental Factors on Spermatogenesis and Sperm Quality

Another topic in our Special Issue, with two research articles, is the influence of environmental factors on spermatogenesis and sperm quality. The relationship between environmental toxins and male fertility has been a focus for decades [7].

Alahwany et al. reported the impact of ultraviolet C radiation on male fertility in rats. Marín de Jesús et al. determined that early zinc supplementation may rescue rats damaged by cadmium exposure. Zn may enhance epididymal sperm glycosylation, endocrine activity, and antioxidant activity in Cd-exposed rats.

### 2.4. Studies That Have Potential Clinical Applications

This Special Issue published three research articles with potential clinical applications.

Sperm selection is one of the key steps for IVF. In the contribution by Ješeta et al., the authors compared swim-up and microfluidic sperm sorting methods for ICSI. The clinical application of this work is high; in normozoospermic patients, both SU and MESS methods can be used for sperm selection, but for non-normozoospermic patients, MFSS is more effective than SU, because MESS may significantly reduce sperm DNA fragmentation.

The second work related to ICSI was contributed by Amor et al. Interleukins and prostaglandins may affect fertilization quality after intracytoplasmic sperm injection (ICSI) procedures. This study revealed the possible influence of interleukins IL-17 and IL-18 and prostaglandins PGE2 and PGF2 on male infertility. The author concluded that IL-18 may play a role in fertilization, and PGE2 may affect embryo development. Further investigations into the molecular mechanisms are needed with animal models.

This Special Issue focuses on sperm quality and fertility. What is the relationship between advancing age and sperm quality? Beg et al. investigated this question at the proteomic level on Arabic men, with ethical approval “11-CEGMR-Bioeth-2020”. They found unique proteins at different ages: MYLK4 was found in the young adult group, PRSS57 in the late adult group, and HMGB4, KRT4, LPGAT1, OXCT2, and MGRN1 in the advanced age group. According to the data, the authors analyzed age-associated alterations that affect sperm fertility: sperm motility, energy metabolism, cellular homeostasis and DNA damage repair, antioxidation, immune response, catabolism, capacitation, acrosome reaction, and fertilization. This study provided basic knowledge for further investigation into sperm fertility and infertility.

## 3. Summary and Perspective

The research in this Special Issue can be further broadened. The morphological and structural observation on sperm of Metazoa in the review article by Guseva et al. may stimulate further research into the sperm evolutionary route in Metazoa. Based on the current advances in spermatogenesis and male infertility, the following topics need further attention:

### 3.1. Structure Formation and Its Molecular Mechanisms During Spermatogenesis Are Very Important

Only a few reproductive biologists work on morphological and structural analysis during spermatogenesis; however, molecular mechanism analysis should be investigated along with morphological observation. Sertoli cells play important roles for spermiogenesis [8,9], and multiple signal pathways are involved in the process of spermatogenesis [10,11]. We also need to focus on how the special ultrastructure of sperm is formed [12,13,14], as well as genome, proteome, and transcriptome approaches; these techniques will help us to understand the activities of genes and proteins during spermatogenesis. Discoveries in these field will improve clinical therapy.

### 3.2. The Hypothalamic–Pituitary–Gonadal Axis Needs Attention

As we know, the hypothalamic–pituitary–gonadal axis plays a vital role in spermatogenesis [1,2]. LH, FSH and their dependent signal pathways are involved in this axis, and disruption of this axis causes male infertility. In-depth knowledge about how this axis works is necessary for clinical diagnosis.

### 3.3. Protection of the Biological Barriers in Testes Needs More Attention

The biological barriers in the testes have been studied for decades, in particular, the blood–testis barrier [3,15,16]. In this Special Issue, Qi et al. reported that the MAPK signaling pathway helps to maintain the integrity of the hemolymph–testis barrier (HTB). HTB exists in invertebrates, especially in crustaceans. If the BTB or HTB are destroyed, spermatogenesis will be abnormal. Environmental factors may affect the blood–testis barrier [17,18]; however, how these biological barriers work still remains obscure.
